# Mutual Effects of Face-Swap Deepfakes and Digital Watermarking—A Region-Aware Study

**DOI:** 10.3390/s25196015

**Published:** 2025-09-30

**Authors:** Tomasz Walczyna, Zbigniew Piotrowski

**Affiliations:** Electronics and Telecommunications Faculty, Military University of Technology, 00-908 Warsaw, Poland; zbigniew.piotrowski@wat.edu.pl

**Keywords:** digital image watermarking, multimedia security, deepfake face swap, generative adversarial networks, invisible watermark, visible watermark, region-aware evaluation, non-local image edits, digital image forensics

## Abstract

**Highlights:**

**What are the main findings?**

- Region-aware evaluation across visible and invisible watermarks with tunable strength and six face-swap families shows that edits are non-local and non-monotonic—background changes introduced by generators even degrade watermarks that are far from the face, and retention does not vary linearly with embedding strength.
- A locality-preserving baseline bounds the minimal impact—architectures that better confine edits to the facial region, typically those with segmentation-weighted objectives, preserve background watermark signal more reliably than globally trained GAN pipelines.

**What are the implications of the main findings?**

- Classical robustness tests for watermarking are not sufficient on their own—evaluation should include generator-induced transformations from face swap and report region-wise metrics for face and background.
- Watermark strength and placement should be selected in an architecture-aware manner—in our sweeps, appropriately tuned invisible marks achieved higher background correlation under manipulation than visible overlays at comparable perceptual distortion.

**Abstract:**

Face swapping is commonly assumed to act locally on the face region, which motivates placing watermarks away from the face to preserve the integrity of the face. We demonstrate that this assumption is violated in practice. Using a region-aware protocol with tunable-strength visible and invisible watermarks and six face-swap families, we quantify both identity transfer and watermark retention on the VGGFace2 dataset. First, edits are non-local—generators alter background statistics and degrade watermarks even far from the face, as measured by background-only PSNR and Pearson correlation relative to a locality-preserving baseline. Second, dependencies between watermark strength, identity transfer, and retention are non-monotonic and architecture-dependent. Methods that better confine edits to the face—typically those employing segmentation-weighted objectives—preserve background signal more reliably than globally trained GAN pipelines. At comparable perceptual distortion, invisible marks tuned to the background retain higher correlation with the background than visible overlays. These findings indicate that classical robustness tests are insufficient alone—watermark evaluation should report region-wise metrics and be strength- and architecture-aware.

## 1. Introduction

Digital image processing and computer vision are increasingly underpinning sensing pipelines, in which images are captured, processed, and authenticated at scale. In this context, multimedia security—including watermarking—intersects with CV tasks such as detection and generative manipulation [1,2,3,4,5,6]. A controlled, region-aware study of two watermark types across six face-swap families, including a locality-preserving baseline, to quantify both identity transfer and watermark retention is conducted.

Beyond achieving this objective, four specific contributions are provided to the multimedia security community:

- A two-sided, region-aware evaluation protocol that quantifies both identity transfer and watermark retention;
- Empirical evidence that generator edits are non-local and that the relationship between watermark strength, identity transfer, and retention is non-monotonic, challenging the common assumption that placing a mark away from the face suffices;
- An architecture-aware analysis showing that methods which better confine edits to the facial region—typically those leveraging segmentation-weighted objectives—preserve background watermark signal more reliably than globally trained GAN pipelines;
- Practical guidance for robustness evaluation in sensing workflows, indicating when tuned invisible marks retain more background correlation than visible overlays at comparable perceptual impact.

Classical watermark robustness studies primarily evaluate compression, resampling, and noise [7,8,9,10], whereas face-swap research focuses on optimizing identity transfer and realism [11,12,13,14,15,16]. At the same time, watermarking has been explored in broader multimedia domains, including 3D cultural heritage protection, where subtle geometric alterations serve as a robust and imperceptible watermarking strategy [17]. Such works highlight the cross-domain importance of balancing imperceptibility, robustness, and usability—challenges that are echoed in the image-based setting studied here. Recent studies have also begun to explore the interaction between watermarking and generative models such as GANs and diffusion networks. For example, adversarial attacks based on generative losses have been shown to reduce watermark readability. At the same time, steganography and color conversion approaches highlight how global changes in image statistics can compromise embedded signals, as also demonstrated by [18], who proposed a concealed attack using generative models and perceptual loss, showing that GAN-based attacks can actively compromise the readability of watermarks. Similarly, diffusion-based pipelines introduce multi-scale transformations that may unintentionally distort watermark energy across both local and background regions. These findings motivate a region-aware analysis of watermark robustness under modern generative transformations. Prior work rarely couples region-aware analysis with visible vs. invisible marks; this study addresses this gap with controlled sweeps on VGGFace2 and a locality baseline [19]. Still images and face swap are taken as the manipulation type, which aligns with common image-centric sensing scenarios.

To enable controlled comparisons, parameterizable variants of the watermarking and face swap methods implemented in experiments. Although other manipulation families (reenactment, lip animation, full-face synthesis) may also affect watermarking, face swap is selected as the primary case study to establish a precise reference and a dedicated baseline for localized edits.

Next, the watermarking and face-swap methods, the region-aware protocol, and the results are outlined, followed by implications for robustness evaluation.

## 2. Materials and Methods

### 2.1. Watermark

A digital watermark is identification information intentionally incorporated into an image, sound, video, or document that remains associated with the file during processing. Its basic functions include confirming the source or owner of the content, tracking its distribution, and, in some systems, user authorization. A watermark can be visible, such as a semi-transparent logo that discourages unauthorized use, or hidden, embedded in the spatial or frequency domain in a way that is invisible to the viewer but can be read after typical editing operations such as compression, scaling, or cropping [10,20].

The key features of a well-designed watermark include: robustness, imperceptibility to the end user, capacity to store additional bits, and security against counterfeiting. In practice, this requires a compromise: the more robust the mark, the greater the interference with the data and the potential deterioration in quality; the more discreet it is, the more difficult it is to ensure its readability after aggressive processing. In the case of publicly published content, a hybrid approach is often employed—a visible logo serves as a deterrent against simple copying. At the same time, a hidden identifier facilitates the enforcement of rights in the event of a dispute [21].

This analysis considers two extreme variants of watermarking—visible and invisible—as they represent two basic content protection strategies, directly noticeable or completely invisible to the end user. The research does not focus on preserving the semantic content encoded in the watermark, but on assessing its resistance to DeepFake-type modifications.

#### 2.1.1. Visible Watermark

The analysis employed an explicit watermark in the form of a QR code spanning the entire frame. This solution ensures the uniform distribution of the mark’s pixels in the image and eliminates the risk of omitting any area when assessing the marking’s impact. During preliminary experiments, alternative visible patterns such as uniform overlays and minor localized marks were also tested. However, these patterns proved less consistent for systematic evaluation: homogeneous marks often interfered with face detection and alignment. In contrast, localized marks were difficult to analyze statistically because their placement did not always overlap with the manipulated region. By contrast, the QR code provides both global coverage and interleaved transparent areas, making it a balanced choice that captures the types of distortions observed with other patterns while remaining analyzable across all scenarios.

**Figure 1:**
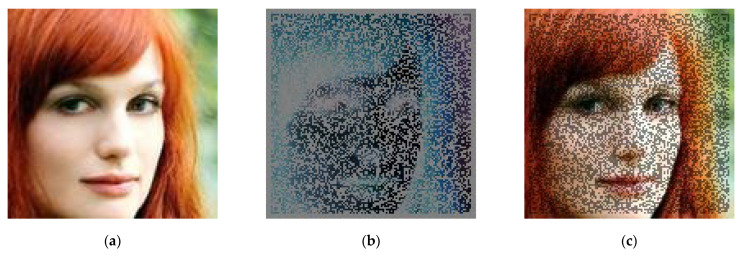
A single example of implemented explicit watermarking: (**a**) image without a watermark, (**b**) difference map (image with watermark minus image without watermark), (**c**) image with watermark (50% opacity).

For clarity, a single example—a synthetic face image—is presented in three variants: (a) reference image without a watermark, (b) the difference between the image with a watermark and the reference image, and (c) a composition of both images with a selected level of transparency (Figure 1a–c). This presentation enables the evaluation of the degree to which an explicit watermark affects the image’s details, even before DeepFake methods are applied.

**Figure 2:**
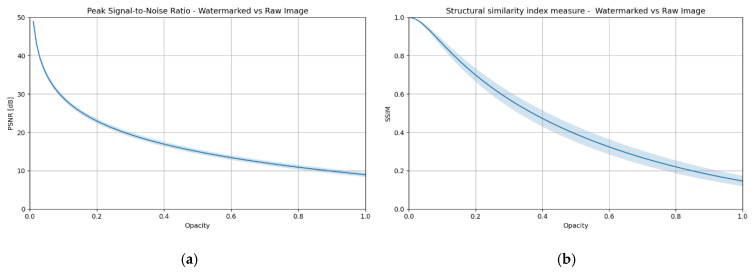
Impact of the transparency level of an explicit watermark on image distortion—comparison of an image with a watermark and a reference image: (**a**) PSNR, (**b**) SSIM.

The impact of transparency on image distortion was determined using two commonly used quality metrics: PSNR (Peak Signal-to-Noise Ratio) and SSIM (Structural Similarity Index) [22]. Figure 2 illustrates the dependence of these metrics on the opacity parameter, which ranges from 0 to 1, corresponding to the level of watermark visibility. The observed curves illustrate a decrease in image quality as the visibility of the mark increases. These graphs serve as a reference point in both subsequent chapters and the experimental part, where the impact of different transparency levels on the effectiveness of DeepFake methods will be analyzed.

#### 2.1.2. Invisible Watermark

The second option analyzed is a hidden watermark embedded using a neural network. Its purpose is to remain completely invisible to the recipient while remaining resistant to typical image processing operations. Many methods of this type have been described in the literature; however, in most cases, it is not possible to precisely control the strength of the interference [7,8]. In studies focused solely on assessing the effectiveness of watermark reading or its impact on a selected task (e.g., classification) [9], such a limitation may be acceptable. However, in a broader analysis—especially when the goal is to generalize the results to an entire group of algorithms (in our case, local face replacement)—it can significantly complicate interpretation.

For this reason, a proprietary model has been developed that allows for smooth adjustment of the watermark signal amplitude—from virtually undetectable to deliberately visible. This allows for a precise examination of the relationship between the strength of the mark and its susceptibility to local modifications, such as DeepFakes.

The designed architecture is based on the classic encoder–decoder approach. The encoder receives an image, to which it matches a watermark, and a message to be embedded. The generated watermark, controlled by the “watermark strength” parameter, is then added to the original image. The resulting image can be manipulated in any way (e.g., face swap), and its degraded version is sent to the decoder, whose task is to recover the encoded message. The invisible watermark used here is purposefully a controllable test instrument rather than a proposed state-of-the-art algorithm. Its novelty for this paper lies in its practicality: the strength parameter is continuously tunable, which enables calibrated sweeps that isolate how watermark energy interacts with generator-induced transforms. This controllability is required to compare visible vs. invisible marks under identical experimental conditions and is not intended as a claim of algorithmic novelty in watermarking.

The encoder was built based on a modified U-Net architecture [23], equipped with FiLM (Feature-wise Linear Modulation) layers [24], which enable the entry of message information at different resolution levels. Residual connections have also been added [25] between successive U-Net levels, which improves gradient flow—a crucial aspect in architectures where part of the cost function is calculated only after the decoder. The encoder output is transformed by a tanh function (with a range of −1 to 1) and then scaled by the “watermark power” parameter (default: 0.1). The default value of the “watermark strength” parameter = 0.1 was adopted experimentally as the midpoint of the range [−1, 1] used in the training process. It ensured a clear yet moderate level of interference with the image, allowing for tests of both greater subtlety and higher visibility of the marker.

To increase the generality of the model and avoid situations where the network hides the watermark only in selected locations, a set of random perturbations was used during training: Gaussian noise, motion blur, Gaussian blur, brightness and contrast changes, resized crop, and random erasing. These were not intended to teach resistance to specific attacks (e.g., face swap) but to force the even distribution of the watermark throughout the image.

The decoder is based on the ResNet architecture [26], whose task is to reduce a tensor containing a degraded image to a vector representing the encoded message.

The learning process involved two primary components of the cost function. The first concerned the correctness of message reading by the decoder:

(1) $$L_{msg}=\;BCEWithLogitsLoss\left(z,\;m\right)$$

where z—output tensor from the decoder, m—coded message.

The second component was responsible for minimizing the visibility of the watermark. For this purpose, a combination of mean square error (MSE) and LPIPS metrics was used [27], better reflecting the difference between images as perceived by humans:

(2) $$L_{vis}=\;MSE\left(\hat{x},\;x\right)+0.2\cdot \;LPIPS(\hat{x},\;x)$$

where $\hat{x}$—image with watermark, x—original image.

All images were scaled to a resolution of 128 × 128 pixels. The adopted resolution of 128 × 128 pixels is lower than typical in practical applications. This limitation resulted from the computational requirements associated with training multiple deepfake networks and a watermarking model in real time, given the available hardware resources. For the comparative analysis, maintaining consistent experimental conditions was prioritized over achieving absolute image resolution. The message length was fixed at 64 bits, generated randomly during training. VGGFace2 [28] served as the dataset, containing photos of different people, which ensured consistency with the rest of the experiments.

In the case of this model, the key indicator in the context of comparative analysis is not the fact that the message was read correctly (full effectiveness was achieved during training), but the impact of the “strength” parameter of the watermark on its actual visibility and level of interference with the image.

**Figure 3:**
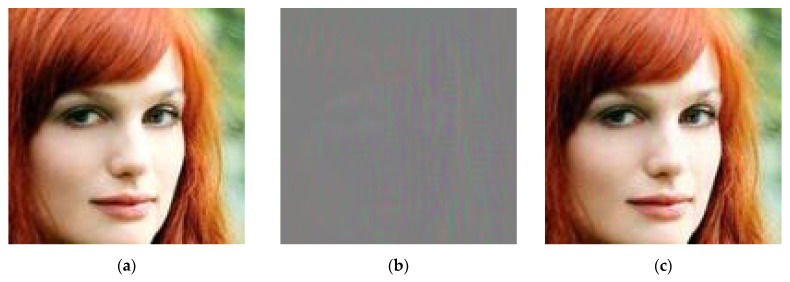
An example of implemented hidden watermarking: (**a**) image without watermark, (**b**) difference map, (**c**) image with watermark (strength 0.1).

**Figure 4:**
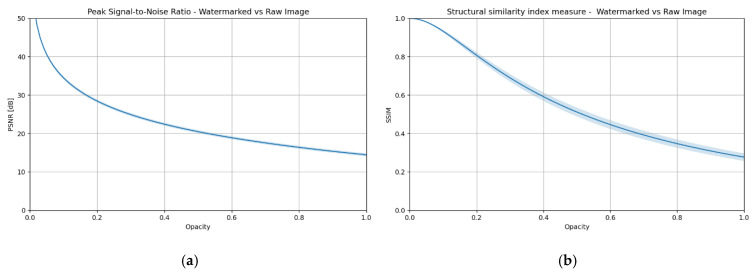
The influence of the “strength” parameter of a hidden watermark on image distortion—comparison of an image with a watermark with the original image: (**a**) PSNR, (**b**) SSIM.

Figure 3 illustrates an example of an image in the default configuration (watermark strength = 0.1) along with its corresponding difference map. Figure 4 illustrates the impact of the “strength” parameter on PSNR and SSIM metrics compared to the original image [8].

### 2.2. Face Swap

Face swap is a class of generative algorithms whose goal is to insert a source face into a target image or video in a way that is believable to a human observer, with no visible traces of modification [11,29]. The typical process includes: face detection and alignment, extraction of its semantic representation (embedding), reconstruction or conditional generation of a new texture, and applying it with a blending mask to the output frame [11].

Although theoretically the modification should be limited to the face area, in practice many models—especially those based on generative adversarial networks (GANs)—also affect the background, lighting, and global color statistics. There are many reasons for this behavior, ranging from the nature of the cost functions used to the specifics and complexity of the model architecture.

In the context of watermarking, this leads to two significant consequences. First, even local substitution can unintentionally distort the signal hidden throughout the image, reducing the effectiveness of invisible watermarking techniques. Second, suppose the visible watermark is located near a face or its pattern resembles an image artifact. In that case, the model may attempt to “correct” it, resulting in reduced legibility of the mark.

For further analysis, popular end-to-end networks such as SimSwap [12] and FaceShifter [13] were selected, as well as newer designs incorporating additional segmentation models, key point generation (Ghost [14], FastFake [15]), or closed solutions—InsightFace [16]. Each of these methods controls the scope of editing differently and achieves a different compromise between photorealism and precise control of the modification region.

In some cases, it was necessary to reimplement or adapt the models to meet the established experiment criteria, which may result in slight differences from the results presented by the authors of the original algorithms. Where possible, the same architectures and cost functions as in the original implementations were retained.

To establish a reference point, a proprietary reference method was also developed, based on classic inpainting in the face segmentation mask area. This method edits only the face region, preserving the background pixels, which allows for estimating the minimal impact of a perfectly localized face swap on the watermark. A comparison of these approaches enables the determination of the extent to which modern, intensely trained models interfere with image content outside the target modification area and allows the theoretical reasons for this behavior to be presented.

While our experiments operate on still images, this setup mirrors the inference mode of many production face-swap pipelines, which process frames independently and compose them into a video. Temporal stabilization (when present) is typically added as a post hoc stage or auxiliary loss and does not alter the core per-frame identity transfer mechanism analyzed in this study. The non-local background effects reported in the results are therefore expected to persist in video, with temporal consistency potentially modulating their magnitude rather than their nature.

#### 2.2.1. SimSwap

SimSwap [12] is one of the first publicly available architectures that enable identity swapping for arbitrary pairs of faces without requiring the network to be retrained. It combines the simplicity of a single encoder–decoder–GAN setup with the ability to work in many-to-many mode.

The key element of SimSwap is the ID Injection module. After encoding the target frame, the identity vector from the source—obtained from a pre-trained ArcFace model [30]—is injected into the deep layers of the generator using Adaptive Instance Normalization (AdaIN) blocks [31].

To preserve the facial expressions, pose, and lighting of the target image, the authors introduced Weak Feature Matching Loss, which compares the deep output representations of the discriminator between the target image and the reference image. This function promotes the consistency of visual attributes by treating the discriminator as a measure of realism rather than identity consistency.

Identity is enforced through a cost function based on the cosine distance between ArcFace embedding vectors. The realism of the generated images is improved by classic hinge-GAN loss and gradient penalty [32]. Additional Reconstruction Loss is activated when the source and target images depict the same person—in this case, the network learns to minimize changes in the image.

In practice, this combination of cost functions means that modifications are concentrated mainly in the face area, while the background and clothing elements remain largely unaffected. However, the lack of an explicit segmentation mask means that subtle color corrections may occur throughout the frame when there are substantial exposure changes or low contrast.

#### 2.2.2. FaceShifter

FaceShifter [13] is a two-stage face swap network designed to preserve the identity of the source without requiring training for each pair. In the first phase (AEI-Net), the following components are combined: an identity embedding obtained from ArcFace [30] and multi-level attribute maps generated by a U-Net encoder.

Integration is achieved using the Adaptive Attentional Denormalization (AAD) mechanism, which dynamically determines whether a given feature fragment should originate from the embedding ID or the attribute maps. In addition to identity loss, adversarial loss, and reconstruction loss, the cost function also uses attribute loss, which enforces attribute consistency between the target image and the replaced image.

The lack of an explicit segmentation mask means that, in cases of significant differences in lighting or color, AAD can also modify the background, which, from a watermarking perspective, increases the risk of distorting the invisible watermark. At the same time, precise attention masks within AAD keep the primary energy of changes within the face.

#### 2.2.3. Ghost

Authors of GHOST [14] presented a comprehensive, single-shot pipeline that covers all stages—from face detection to generation and super-resolution. However, only the GAN core is relevant in the context of this analysis. The basic architecture is a variation in AEI-Net known from FaceShifter, but with several significant modifications.

Similarly to FaceShifter, the identity vector obtained from the ArcFace model is injected into the generator using Adaptive Attentional Denormalization (AAD) layers. A new feature is the use of an additional network targeting the eye region, along with a redesigned cost function—specifically, eye loss—which enables the stable reproduction of gaze direction in the generated image.

The second improvement is the adaptive blending mechanism, which dynamically expands or narrows the face mask based on the differences between the landmarks of the source and target images. This solution enhances the fit of the face shape and edges, thereby increasing the realism of the generated image.

In this study, the adaptive blending and super-resolution stages were omitted to focus solely on the analysis of pixel destruction introduced by the generator itself. Furthermore, some of the elements introduced in GHOST, such as adaptive blending, are not differentiable, which could disrupt the training process if a labeling model is to be used, treating face swaps as noise in the learning process.

#### 2.2.4. FastFake

Fast Fake [15] is one of the newer examples of a lightweight GAN-based face swap, where the priority is fast and stable learning on small datasets, rather than achieving photographic perfection in each frame. The core of the model is a generator with Adaptive Attentional Denormalization (AAD) blocks, borrowed from FaceShifter [13]. Still, the entire architecture was designed in the spirit of FastGAN [33], featuring fewer channels, a skip-layer excitation mechanism [34], and a discriminator capable of reconstructing images, which helps limit the phenomenon of mode collapse.

The key difference from the previously discussed models lies in the way segmentation is utilized. The authors include a mask from the BiSeNet network [35] only at the loss calculation stage—pixels outside the face area are sent to the reconstructive MSE, and features obtained from the parser are additionally blurred and compared with analogous maps of the generated image. As a result, the generator learns to ignore the background, because any unjustified change in color increases the loss value. During inference, the mask is no longer used, keeping the computation flow clean and fast.

From the perspective of analyzing the impact of DeepFake on watermarking, this approach has significant implications. The scope of FastFake interference is even narrower than in SimSwap or FaceShifter—global color statistics change minimally, which potentially favors the protection of watermarks placed outside the face area. In theory, the GAN cost function should interfere to some extent with the component that enforces background preservation. However, it cannot be ruled out that the generator will still harm unusual elements of the image, such as watermarks.

Thanks to its low data requirements and fast learning process, FastFake is a representative example of the “economical” branch of face swap methods, which will be compared with other models in terms of their impact on the durability and legibility of watermarks later in this article.

#### 2.2.5. InsightFace

The InsightFace Team [16] has not published a formal article describing the Inswapper module; however, this model is widely used in open-source tools, including Deep-Live-Cam [36], and functions as an informal “market standard” in the field of face swapping.

Similarly to the previously discussed methods, Inswapper uses a pre-trained ArcFace model to determine the target’s identity. Although the implementation details are not fully known, a significant difference is the surrounding pipeline: InsightFace provides a complete SDK with its own face detection module and predefined cropping, which also includes arms and a portion of the background. If the detector does not detect a face or rates its quality below a certain threshold, the frame remains unchanged. In the context of watermarking, this means that elements outside the detected face mask can remain completely intact. This feature is also valuable for experiments—it allows the assessment of whether the degradation of the watermark is significant enough to prevent the image from being used by popular face swap algorithms.

For this paper, the analysis is limited to the generator block and the mandatory face detector, omitting subsequent stages of the pipeline, such as skin smoothing and super-resolution. In this context, Inswapper serves as a realistic but minimal attack: any violation of the watermark is solely the result of identity substitution—provided that the face is detected and passed on for processing—which reflects a typical use case in popular consumer tools.

#### 2.2.6. Baseline

The last face swap algorithm analyzed is a proprietary reference method explicitly developed for this comparison. Although it does not achieve SOTA results on its own, it stands out with its local face replacement range and an interesting approach to separating information from sources of the same type. Unlike the previously discussed GAN models, the training process uses elements characteristic of currently popular diffusion models [37].

The algorithm consists of three main components:

- U-Net—typical for diffusion models, responsible for removing the noise.
- Identity encoder—compresses input data into a one-dimensional hidden space; receives a photo of the same person, but in a different shot, pose, or lighting.
- Attribute encoder—also compresses data into a hidden space, but accepts the target image in its original form.

At the input, U-Net receives an image with a noisy face area (following the diffusion model approach) and a set of conditions: noise level, attribute vector, and identity vector.

The goal of the model is to recreate the input image based on additional information provided through conditioning. During inference, when a face of another person is fed to the identity encoder, the U-Net generates an image with the identity swapped, while preserving the pose, facial expressions, and lighting resulting from the attribute vector.

The key challenge is to motivate the model to utilize information from the identity encoder, rather than solely from the attribute encoder, which, in the absence of constraints, could contain all the data necessary for reconstruction. To prevent this, three modifications to the attribute vector were applied:

- Masking—randomly zeroing fragments of a vector, which forces the model to draw information from the identity encoder, as they may be insufficient on their own.
- Dropout—increases the dispersion of information in the vector, preventing data concentration in rarely masked fragments.
- Normal distribution constraint (KL divergence loss)—inspired by the VAE approach [38]; forces the elements of the attribute vector to carry a limited amount of information about the details of a specific image.

Thanks to these modifications, the model distributes information more evenly in the hidden space and obtains most of the identification features from the identity encoder.

During inference, both the noise step (typical for diffusion models) and the masking level of the attribute vector can be controlled. Only one diffusion step was applied in the study—subsequent steps would improve the visual quality of the face swap without significantly affecting the hidden watermark.

Masking levels within the Baseline method were selected based on a series of preliminary tests with a trained network. The parameters were selected to ensure that the effect of masking on the extent of image modification was subjectively visible, while maintaining a comparable quality of the generated face. This differentiation enabled the analysis of how varying degrees of attribute isolation impact the degradation of the watermark.

The ability to control the noise level enables the generation of multiple test samples for various initial settings. In addition to analyzing the impact on the watermark, this approach can be used to augment training data for face-swap-resistant tagging systems.

#### 2.2.7. Examples of Implemented Deepfakes

**Figure 5:**
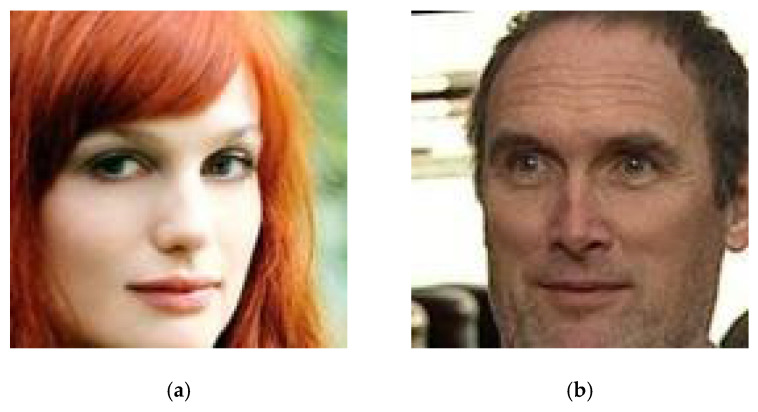
Reference images used in unit and comparative tests: (**a**) target image, (**b**) source image.

Images from the VGGFace2 [28] dataset were used for unit testing, shown in Figure 5:

(a) Image of the target face—the one that will be replaced,
(b) Source identity for face swap algorithms.

Table 1 presents the sample face replacement results obtained for all models discussed (SimSwap, FaceShifter, GHOST, FastFake, InsightFace, and Baseline), along with heat maps illustrating the changes made. For the baseline model, three variants are presented, corresponding to different levels of attribute vector masking (high, medium, low).

The table shows that some deepfake algorithms also introduce changes in the background of the image. This is particularly evident in the SimSwap and FaceShifter methods, where the right side of the heat maps indicates significant modifications outside the face area.

### 2.3. Experiments

Several research scenarios were conducted as part of the analysis. Due to the intertwining nature of the individual experiments, they were divided into two main blocks: visible tagging and hidden tagging. Each block broadly covers analogous types of analysis, specifically examining the impact of tagging on face swapping on a global scale, broken down into face and background areas.

First, the results for visible tagging will be presented, followed by those for hidden tagging. In both cases, individual examples of the impact of tagging with different parameter configurations are presented, followed by a statistical overview based on a test set.

The primary metrics used in the analyses are the following:

- ArcFace and CurricularFace [39] distance—the cosine distance between feature vectors extracted by the models, allowing for assessing whether the persons depicted in the compared images are recognized as the same.
- Pearson correlation—Pearson correlation coefficient between the image with the watermark and the image after applying face swap to the material containing the watermark.
- PSNR (Peak Signal-to-Noise Ratio)—used additionally in local analyses for a selected area (background).

The study analyzed two fundamental aspects:

The impact of watermarks on face swaps:

- Heatmaps of differences between the image after face swap performed on material with a watermark and the image after face swap performed on material without a watermark were compared.
- The ArcFace and CurricularFace distance between these two variants was calculated to assess the impact of marking on identity recognition.
- Watermark retention:
- The Pearson correlation coefficient was calculated between the original image with the watermark and the image after face swapping was performed on the same image.
- Correlation maps and heat maps showing the distribution of changes in the image were generated.
- In local analyses, the background area was examined separately by calculating the PSNR for this region to estimate the impact of face swap outside the face area.

All experiments were performed on the VGGFace2 dataset. The VGGFace2 dataset includes photographs with varying lighting conditions, quality, compression, and cropping, which allowed the natural incorporation of the diversity typical of images obtained from the web into the experiments. VGGFace2 was chosen because it is widely used to train the face-swap families evaluated in this study, ensuring consistency between the training distribution of the generators and the test distribution of the watermarking experiments. Using a different dataset could have introduced additional variability due to distribution shifts, potentially confounding the interactions between the watermark and the generator. The focus was therefore on establishing controlled, region-aware comparisons under conditions representative of mainstream face-swap pipelines. Extending the evaluation to additional datasets, including those with different demographics and capture conditions, remains an important direction for future work to assess generalizability further. For each combination of parameters, calculations were performed on 1000 examples, allowing for statistically significant comparisons. The results are compared with a local baseline, which allows the quantitative demonstration of the non-locality of editing outside the face area.

## 3. Results

### 3.1. Visible Watermark

This section presents the results for a visible watermark, unrelated to any neural network training process, and its interaction with deepfake algorithms.

#### 3.1.1. The Impact of Watermarks on the Face Swap Algorithm

The first study analyzed the impact of introducing a visible watermark on the performance of face swap algorithms, both in terms of generation quality and differences compared to the unmarked variant. Since this is a visible mark, a natural effect is a decrease in metrics such as PSNR as the opacity parameter increases.

Table 2 presents examples illustrating the differences between images after face swapping is performed on material with and without a watermark, along with corresponding heatmaps that show areas of change depending on the opacity value. In this section, the focus is on the face region. Differences in the heatmaps inside the mask indicate whether the visible mark (QR pattern) interferes with identity transfer or whether the generator collapses. A firmly structured QR pattern acts like an occlusion; if its imprint is clearly visible in the face area after swapping, the method either learned to “correct” occlusions in a specific way or partially failed to perform a stable swap. By contrast, a more uniform heatmap confined to facial boundaries suggests that the method edits locally without amplifying the mark.

The qualitative patterns group the methods. SimSwap, FaceShifter, and GHOST show larger, face-internal differences that grow with opacity, which means the watermark perturbs their swap pipeline and can trigger failures (including mode collapse in some settings). FaceShifter is the most sensitive: identity consistency drops already for small occlusions, consistent with its reconstruction/attribute losses—these favor realism but can overfit and become brittle when the face is partially masked. GHOST is strongly tied to ArcFace-style identity losses, which helps enforce identity but also makes it prone to “forcing” identity while drifting the background. FastFake and InsightFace behave differently: FastFake tends to preserve non-face regions (segmentation-weighted objective during training) and confines edits, whereas InsightFace often avoids modifying low-quality/occluded faces because the detector rejects them, so at high opacity, the frame can remain unchanged. The Baseline is intentionally invasive inside the face mask and therefore “cuts through” the watermark in that region while leaving the background intact; it serves as the lower-bound reference for locality.

**Figure 6:**
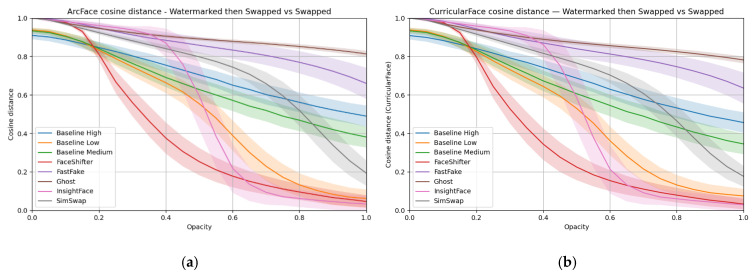
ArcFace (**a**) and CurricularFace (**b**) cosine distance between the face-swapped image with a watermark and the face-swapped image without a watermark.

Figure 6 presents the results in the form of a numerical metric—the cosine distance between feature vectors obtained from the ArcFace and CurricularFace models. A higher value of this metric means greater identity similarity between images. Results from CurricularFace closely mirror those of ArcFace; for brevity, ArcFace curves are discussed, and it is noted that CurricularFace leads to the same qualitative conclusions.

Overall, the joint reading of Table 2 (spatial differences) and Figure 6 (identity distance) is essential: heatmaps reveal where the watermark perturbs the pipeline (i.e., face vs. background), while the identity metric shows how these perturbations translate into recognition outcomes.

#### 3.1.2. Watermark Resistance

**Figure 7:**
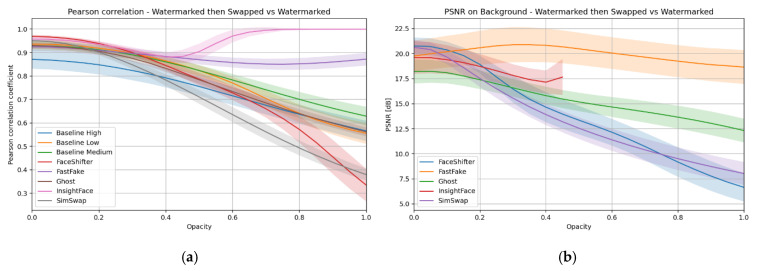
(**a**) Pearson correlation, (**b**) PSNR background between the image with the watermark and the image after face swap—visible watermark case.

Another analysis concerned the behavior of the watermark after processing by face swap. The Pearson correlation between the original image with the watermark and the image after face swap was measured (Figure 7a). A high correlation value indicates that a significant portion of the image remained consistent, and the watermark was preserved.

Table 3 supplements these graphs with visual correlation maps and heatmaps. In the upper rows, colors represent local Pearson correlation: dark red indicates perfect correlation (watermark preserved), while cooler or lighter tones indicate reduced similarity. In the lower rows, the heatmaps show pixel-level differences: colder areas mean fewer changes, while warmer areas highlight more substantial modifications.

An ideal outcome would combine high correlation in the background (dark red outside the face) with visible changes only in the swapped region, similar to the Baseline. In such a case, the watermark is preserved outside the swapped face while the face is modified as expected. For example, at medium opacity (e.g., 20%), FastFake’s maps clearly reveal facial outlines in the correlation map. Still, the rest of the image remains dark red—indicating that the watermark survives in the background.

In practice, deviations from this “ideal” appear. SimSwap, FaceShifter, and Ghost exhibit broader color variation in the upper rows and warmer areas in the lower rows, which extend into the background. This indicates that watermark information is degraded even in regions not directly edited, which is consistent with their training setup (no explicit segmentation or background-preservation loss). InsightFace is distinct: at low opacity, it behaves similarly to SimSwap; however, once the opacity exceeds approximately 40%, the detector rejects the face, and no swap is performed. Therefore, the PSNR for those images is infinity. This results in the truncated red line in Figure 7b: at 75% and 100% opacity, the images remain unchanged, yielding artificially high correlation and PSNR values.

Figure 7b further highlights differences between methods. The baseline remains constant because it only modifies the face area, leaving the background unchanged, so it is not included. FastFake achieves the highest background PSNR among GAN-based models, confirming its ability to preserve non-face areas. SimSwap and FaceShifter consistently degrade background quality, while Ghost exhibits intermediate behavior. InsightFace is truncated for the reason explained above.

Overall, Table 3 and Figure 7 should be interpreted jointly. Strong results appear as dark red correlation in the background with localized warmer areas in the face (Baseline, FastFake). Poorer results appear as irregular, widespread variation in both rows (SimSwap, FaceShifter, Ghost), where the watermark is degraded across the whole frame.

### 3.2. Hidden Watermark

This section presents the results for a watermark trained using a neural network, designed to remain invisible to the viewer.

#### 3.2.1. The Impact of Watermarks on the Face Swap Algorithm

As in the case of visible watermarks, the first stage of the analysis involved comparing the impact of introducing a hidden watermark on the performance of face swap algorithms. Table 4 presents example heatmaps illustrating the differences between images after face swap with and without a hidden watermark. Unlike the visible case, the hidden watermark does not produce a clear visual pattern; therefore, the distortions are subtler and must be interpreted in conjunction with the identity metrics.

**Figure 8:**
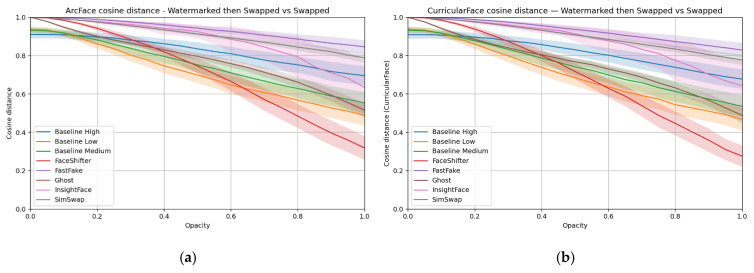
ArcFace (**a**) and CurricularFace (**b**) cosine distance between the face-swapped image with a watermark and the face-swapped image without a watermark—hidden watermark case.

Figure 8 reports cosine distance for ArcFace and CurricularFace embeddings. The two metrics are consistent. Higher values indicate stronger identity preservation.

Several patterns emerge when reading Table 4 and Figure 8 together. SimSwap and FaceShifter maintain identity transfer more consistently than in the visible case and do not collapse in the unit examples. However, their heatmaps still show non-local changes extending into the background, which reduces correlation and partly explains fluctuations in the metric curves. GHOST behaves differently: here, the watermark induces global degradation, visible as widespread heatmap differences, and its identity curve drops accordingly. This reflects its strong dependence on ArcFace-style identity losses, which can compromise stability in favor of identity.

FastFake again shows the most localized edits. In Table 4, the heatmaps reveal clearer preservation of the background compared to other GAN families, with distortions mostly confined to facial features. Interestingly, its ArcFace metric reaches relatively high values even when subjective quality suggests identity loss, highlighting a gap between numerical embeddings and visual perception. This indicates that background preservation (beneficial for watermark retention) can sometimes distort identity embeddings, creating a mismatch between subjective human judgment and model-based evaluation.

InsightFace differs from the visible case: since the hidden watermark does not strongly occlude facial regions, the detector continues to operate across the opacity range. Its identity metric gradually declines, possibly reflecting detector uncertainty, but no abrupt failure is observed.

In summary, hidden watermarks are less likely to induce catastrophic failures than visible ones. Still, the trade-off shifts: methods such as FastFake preserve the background more effectively (favoring watermark survival) but may produce ArcFace distances that overestimate identity consistency. SimSwap and FaceShifter remain more sensitive to background distortions, while GHOST is most vulnerable to global degradation.

#### 3.2.2. Watermark Resistance

**Figure 9:**
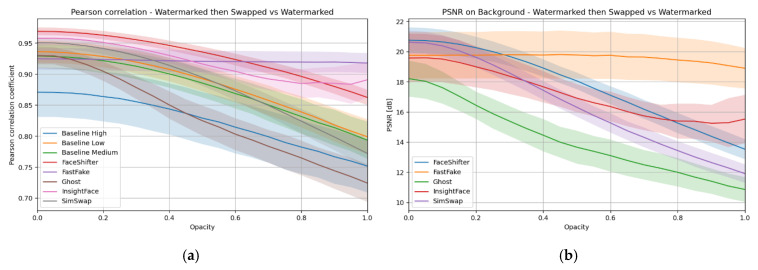
(**a**) Pearson correlation, (**b**) PSNR of the background between the image with the watermark and the image after face swap—hidden watermark case.

As in the case of the visible mark, the resistance of the hidden watermark to face swap processing was assessed using Pearson correlation and background-only PSNR. Figure 9 shows the global trends, while Table 5 provides spatial detail through correlation maps (upper rows) and heatmaps of differences (lower rows). In the correlation maps, similar to before, dark red indicates perfect similarity (with the watermark preserved), while cooler tones signal degraded correlation. In the heatmaps, colder regions indicate small changes, while warmer regions highlight more significant modifications.

The ideal outcome is similar to the visible case: high background correlation with localized changes confined to the swapped face. FastFake comes closest to this behavior. At moderate opacity levels (e.g., 20–30%), its maps clearly show facial outlines but preserve most of the background as dark red, meaning the hidden mark survives outside the swap region.

SimSwap and FaceShifter behave less favorably. Their correlation maps reveal widespread cooler areas that extend into the background, suggesting that the hidden watermark is degraded globally rather than just in the manipulated region. This is consistent with their training setups, which lacked explicit segmentation-based objectives to constrain edits.

GHOST exhibits the most severe background degradation. Its heatmaps display strong warm patches across the frame, showing that the generator alters even regions untouched by the swap mask.

InsightFace shows a different pattern. Unlike in the visible case, it operates across the entire opacity range, so its curves in Figure 9 remain continuous. Its correlation maps reveal that the method detects and processes a slightly different area, which affects interpretation. Still, its behavior is relatively stable compared to SimSwap or GHOST.

Overall, the combination of Table 5 and Figure 9 confirms that FastFake provides the most localized interference, closer to the Baseline, while SimSwap, FaceShifter, and especially GHOST spread degradation across the image. InsightFace remains stable but reflects the influence of its detector. These findings reinforce that the survival of hidden watermarks depends strongly on the architecture: methods with segmentation-aware objectives or detector-based constraints tend to preserve the background signal more effectively, while globally trained GANs degrade it more widely.

## 4. Discussion

The results indicate that invisible watermarks, when trained for robustness to common degradations, generally retain more of their signal after face swap than visible marks. Importantly, the dominant effect is not confined to the face region. Several families of face-swap models introduce non-local changes that also degrade watermarks placed far from the manipulated area. This challenges the common assumption that placing a mark away from the face is sufficient for protection. Dependencies between watermark strength, retention, and identity transfer are also non-monotonic and architecture-dependent. In some models, stronger marks simultaneously harm both transfer quality and retention, while in others, weaker marks are unintentionally smoothed out. Segmentation-weighted models better confine edits to the face and therefore preserve background watermarks more reliably, similar to how robust feature learning improves parsing in complex scenes [40].

These findings refine the evaluation of robustness. Classical tests, such as blur, noise, or resampling, approximate only part of the distortions caused by modern generators. Nonlinear, model-specific transforms produce irregular behaviors that monotonic assumptions cannot capture. Robustness evaluation must therefore be region-aware, strength-aware, and architecture-aware. Allocating watermark energy near facial boundaries or with high contrast carries a high risk of being reinterpreted as artifacts by the generator. Likewise, tuning strength requires careful consideration to avoid detector rejection or global statistical shifts.

This study has limits that point to clear directions for future work. The analysis focused on still images at a resolution of 128 × 128 to keep training multiple face-swap families and the watermarking model computationally feasible. Many pipelines in practice operate at 256 × 256 or higher and often incorporate super-resolution or enhancement modules, which can also affect watermark retention. Extending the experiments to higher resolutions, as well as to video, will help test whether the same non-local effects persist under more realistic conditions. In videos, temporal consistency could either help recovery by exploiting correlations across frames or further suppress weak marks by enforcing smoothness. Other manipulation families, such as reenactment, lip-sync, or full-face synthesis, should also be investigated. Broader datasets, additional recognition backbones, and recovery metrics such as bit error rate (BER) under dedicated training with specific model-induced degradations would also be valuable to investigate. Since the invisible watermark was not trained against face-swap transformations, the BER evaluation in the present setup would fail trivially. Future work should therefore couple BER analysis with watermark models explicitly trained for resistance to deepfake-induced perturbations.

For practitioners, the most actionable heuristic is to train watermarking systems with deepfake generators explicitly modeled as perturbations. In practice, this means augmenting training with generative noise or embedding a generator-in-the-loop, so that the decoder learns to recover messages under realistic non-local edits rather than only under classical distortions. Simple placement strategies such as “away from the face” are not sufficient. The results show that robustness depends strongly on the generator family: segmentation-weighted architectures preserve background signal better, while globally trained GANs often induce drift across the whole frame. Beyond the swap itself, additional stages such as super-resolution and enhancement can introduce comparable degradations. Robustness should therefore be validated across the entire pipeline rather than only the identity transfer step.

Deepfake generation and digital watermarking sit at the intersection of multimedia security, intellectual property protection, and public trust in visual media. While our contribution is methodological, the underlying technologies can be misused for disinformation, identity abuse, or unauthorized redistribution. At the same time, watermarking provides a means to enhance attribution and accountability. It is therefore emphasized that research in this area should be accompanied by guidelines for responsible use, including transparency in reporting, restrictions on dataset access, and a clear separation between forensic evaluation and generative enhancement.

Finally, even lightweight architectures such as FastFake, which appear perceptually stable to the human eye, introduced measurable statistical shifts in the analysis. This shows that human inspection alone is insufficient. Future work should aim to establish standardized, region-aware benchmarks that integrate deepfake generators into robustness testing pipelines. Such benchmarks would ensure that watermarking methods are evaluated under realistic adversarial conditions and would guide practitioners toward generator-aware placement and strength strategies.

## Figures and Tables

**Table 1:** Sample face swap results and corresponding heat maps for the tested models.

	SimSwap	FaceShifter	Ghost	FastFake	InsightFace	Baseline High Mask	Baseline Medium Mask	Baseline Low Mask
Face Swapped	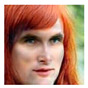	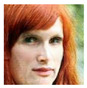	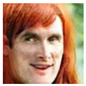	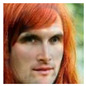	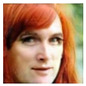	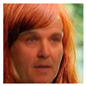	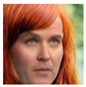	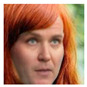
Heatmap	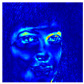	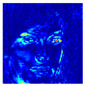	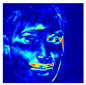	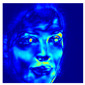	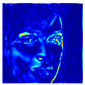	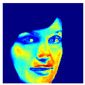	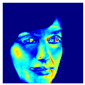	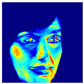

**Table 2:** Heatmaps showing differences between images after face swap with a watermark and images after face swap without a watermark—visible watermark case.

	Image with Watermark	SimSwap	Face Shifter	Ghost	Fast Fake	Insight Face	Baseline High Mask	Baseline Medium Mask	Baseline Low Mask
5%	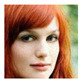	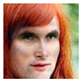	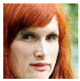	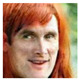	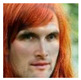	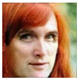	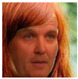	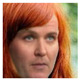	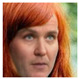
		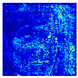	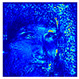	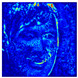	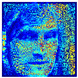	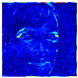	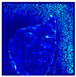	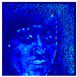	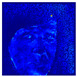
10%	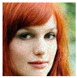	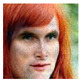	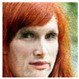	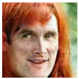	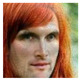	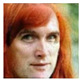	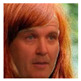	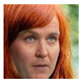	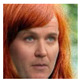
		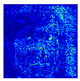	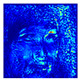	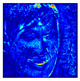	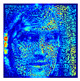	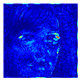	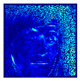	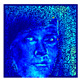	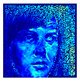
20%	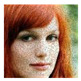	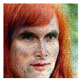	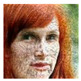	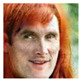	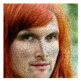	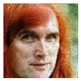	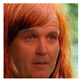	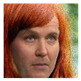	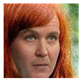
		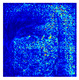	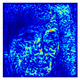	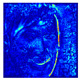	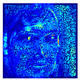	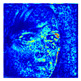	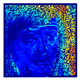	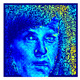	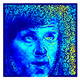
30%	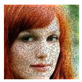	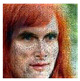	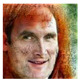	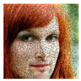	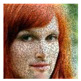	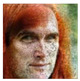	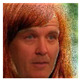	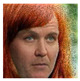	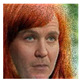
		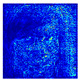	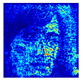	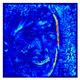	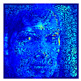	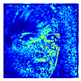	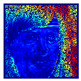	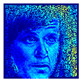	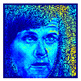
50%	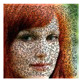	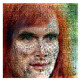	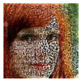	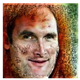	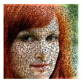	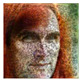	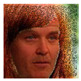	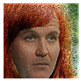	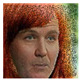
		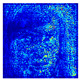	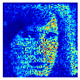	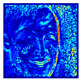	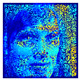	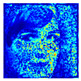	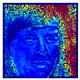	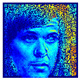	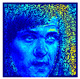
75%	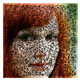	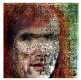	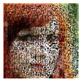	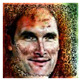	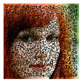	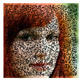	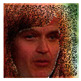	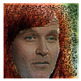	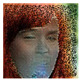
		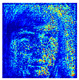	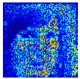	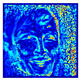	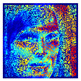	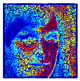	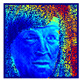	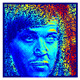	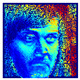
100%	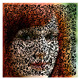	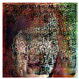	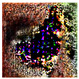	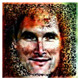	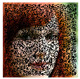	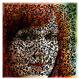	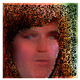	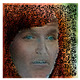	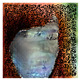
		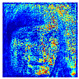	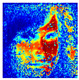	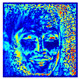	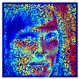	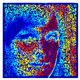	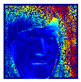	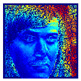	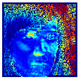

**Table 3:** Local Pearson correlation and heatmaps of differences between the image with the watermark and the image after face swap—visible watermark case.

	SimSwap	Face Shifter	Ghost	Fast Fake	Insight Face	Baseline High Mask	Baseline Medium Mask	Baseline Low Mask
0%	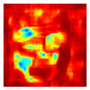	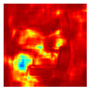	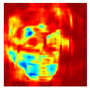	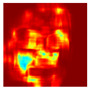	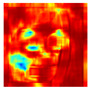	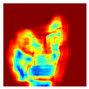	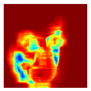	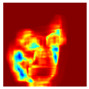
	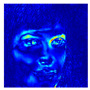	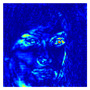	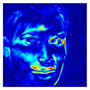	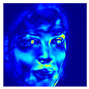	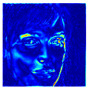	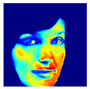	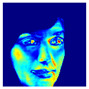	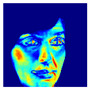
5%	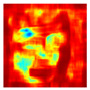	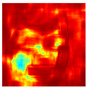	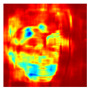	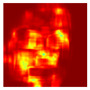	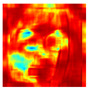	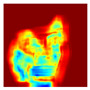	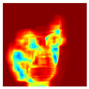	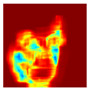
	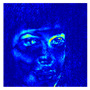	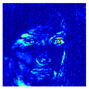	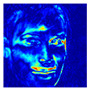	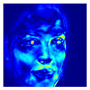	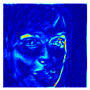	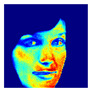	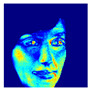	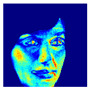
10%	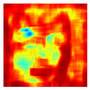	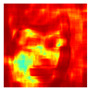	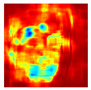	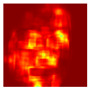	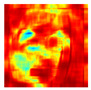	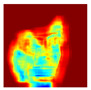	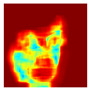	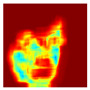
	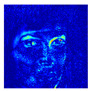	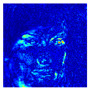	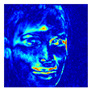	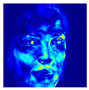	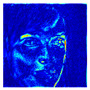	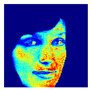	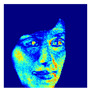	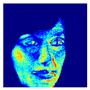
20%	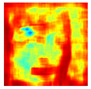	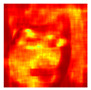	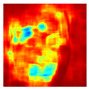	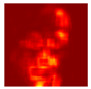	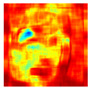	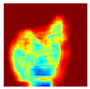	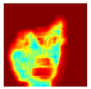	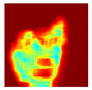
	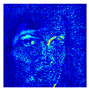	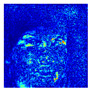	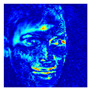	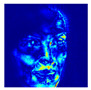	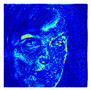	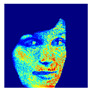	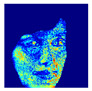	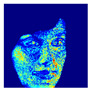
30%	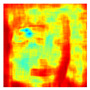	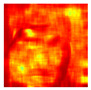	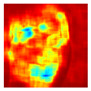	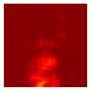	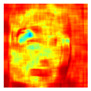	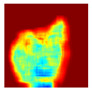	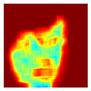	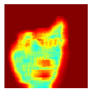
	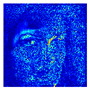	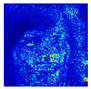	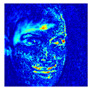	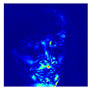	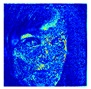	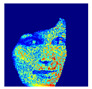	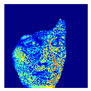	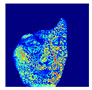
50%	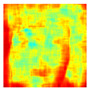	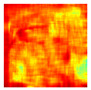	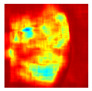	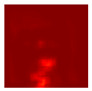	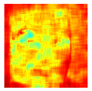	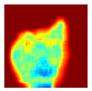	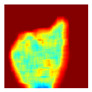	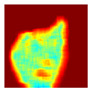
	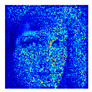	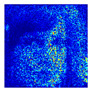	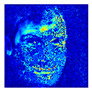	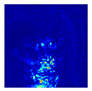	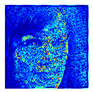	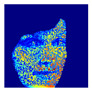	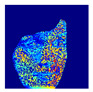	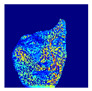
75%	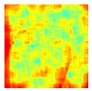	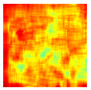	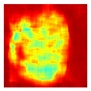	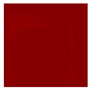	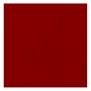	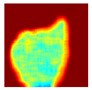	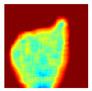	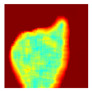
	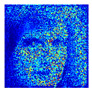	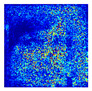	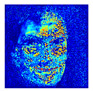	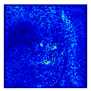	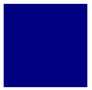	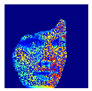	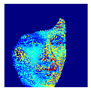	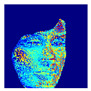
100%	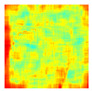	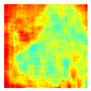	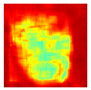	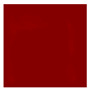	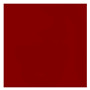	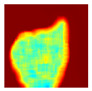	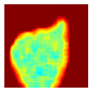	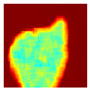
	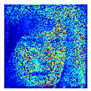	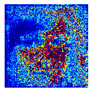	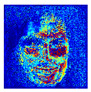	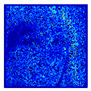	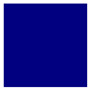	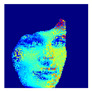	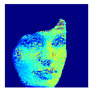	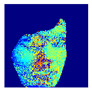

**Table 4:** Heatmaps of differences between images after face swap with a watermark and images after face swap without a watermark—hidden watermark case.

	Image with Watermark	SimSwap	Face Shifter	Ghost	Fast Fake	Insight Face	Baseline High Mask	Baseline Medium Mask	Baseline Low Mask
5%	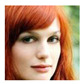	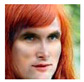	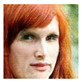	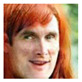	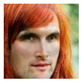	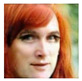	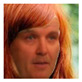	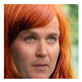	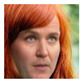
		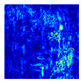	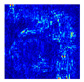	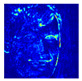	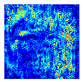	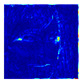	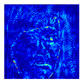	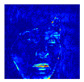	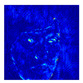
10%	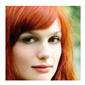	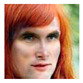	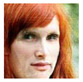	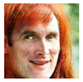	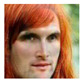	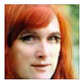	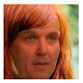	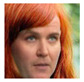	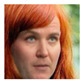
		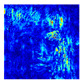	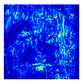	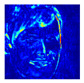	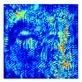	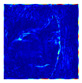	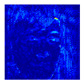	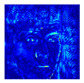	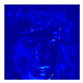
20%	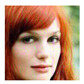	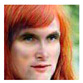	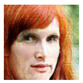	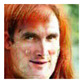	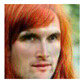	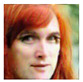	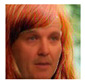	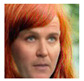	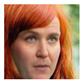
		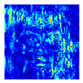	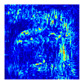	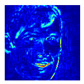	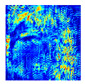	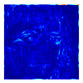	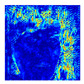	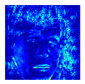	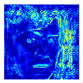
30%	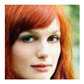	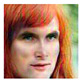	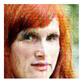	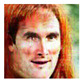	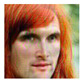	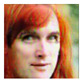	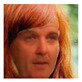	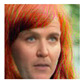	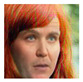
		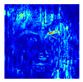	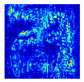	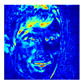	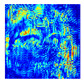	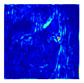	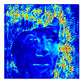	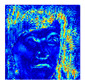	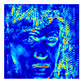
50%	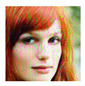	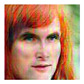	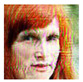	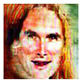	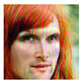	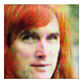	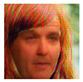	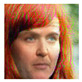	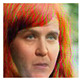
		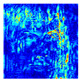	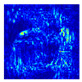	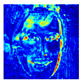	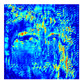	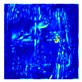	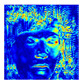	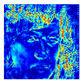	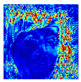
75%	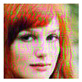	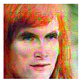	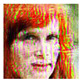	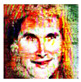	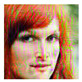	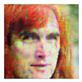	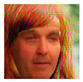	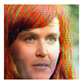	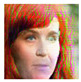
		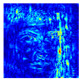	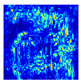	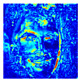	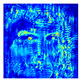	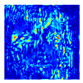	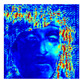	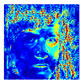	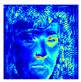
100%	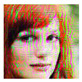	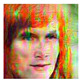	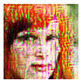	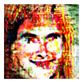	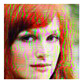	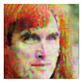	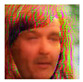	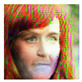	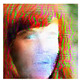
		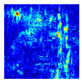	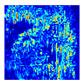	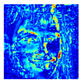	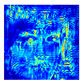	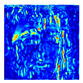	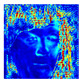	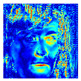	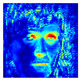

**Table 5:** Local Pearson correlation and heatmaps of differences between the image with the watermark and the image after face swap—hidden watermark.

	SimSwap	Face Shifter	Ghost	Fast Fake	Insight Face	Baseline High Mask	Baseline Medium Mask	Baseline Low Mask
0%	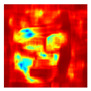	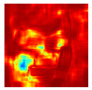	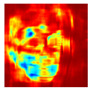	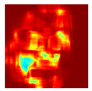	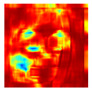	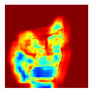	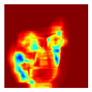	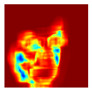
	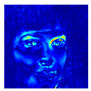	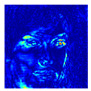	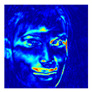	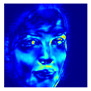	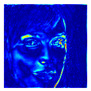	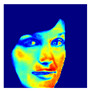	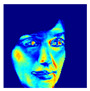	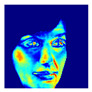
5%	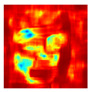	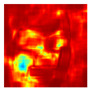	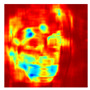	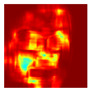	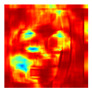	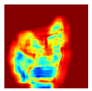	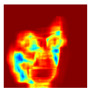	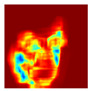
	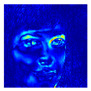	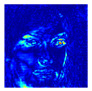	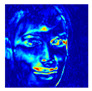	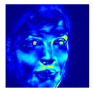	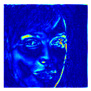	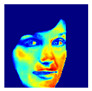	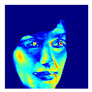	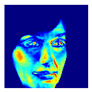
10%	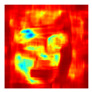	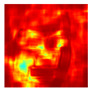	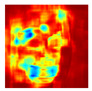	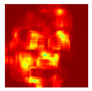	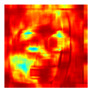	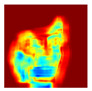	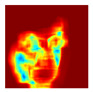	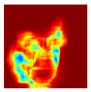
	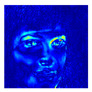	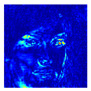	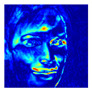	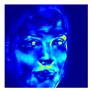	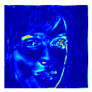	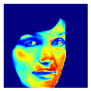	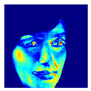	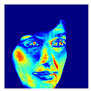
20%	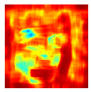	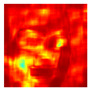	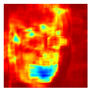	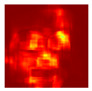	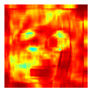	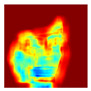	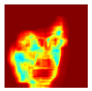	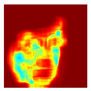
	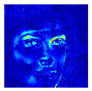	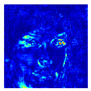	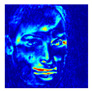	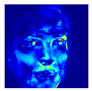	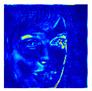	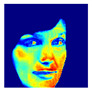	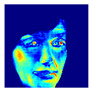	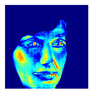
30%	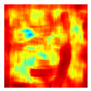	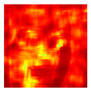	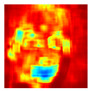	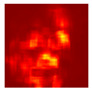	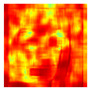	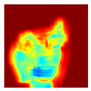	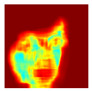	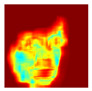
	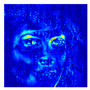	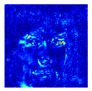	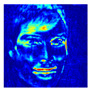	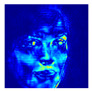	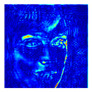	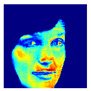	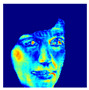	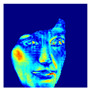
50%	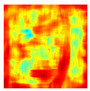	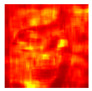	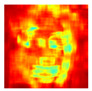	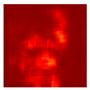	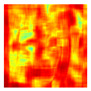	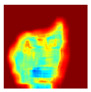	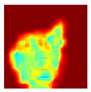	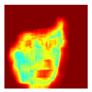
	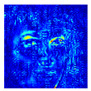	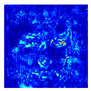	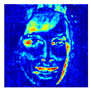	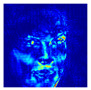	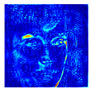	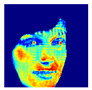	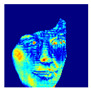	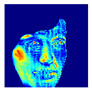
75%	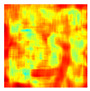	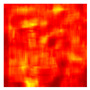	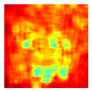	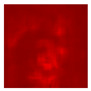	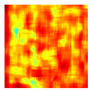	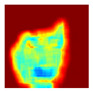	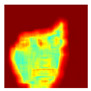	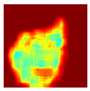
	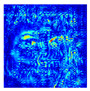	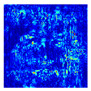	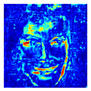	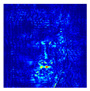	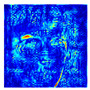	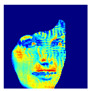	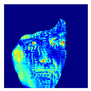	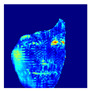
100%	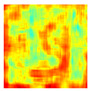	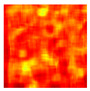	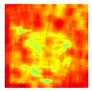	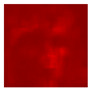	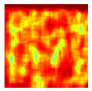	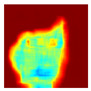	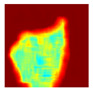	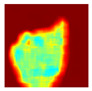
	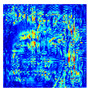	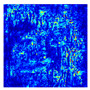	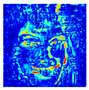	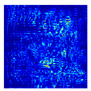	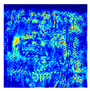	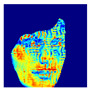	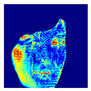	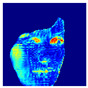

## Data Availability

The data presented in this study are available on request from the corresponding author. The dataset underlying the experiments is VGGFace2, which the authors cannot redistribute due to license terms. Upon reasonable request, we will provide per-image and aggregated metrics, lists of VGGFace2 image identifiers used, configuration files and scripts to reproduce the experiments, and trained checkpoints of the baseline watermarking and face-swap components developed in this work, subject to third-party license restrictions.

## Abbreviations

The following abbreviations are used in this manuscript:

AAD	Adaptive Attentional Denormalization
AdaIN	Adaptive Instance Normalization
AEI-Net	Adaptive Embedding Integration Network
ArcFace	Additive angular margin face recognition model
BCE	Binary Cross-Entropy
BCEWithLogitsLoss	Binary Cross-Entropy with logits
BiSeNet	Bilateral Segmentation Network
CV	Computer Vision
DIP	Digital Image Processing
FiLM	Feature-wise Linear Modulation
GAN	Generative Adversarial Network
ID	Identity embedding
KL	Kullback–Leibler divergence
LPIPS	Learned Perceptual Image Patch Similarity
MSE	Mean Squared Error
PSNR	Peak Signal-to-Noise Ratio
QR	Quick Response code
ResNet	Residual Network
SDK	Software Development Kit
SSIM	Structural Similarity Index Measure
SOTA	State of the Art
U-Net	U-shaped convolutional neural network
VAE	Variational Autoencoder
VGGFace2	VGG Face dataset (version 2)
